# Common Types of Falls in the Elderly Population, Their Associated Risk Factors and Prevention in a Tertiary Care Center

**DOI:** 10.7759/cureus.14863

**Published:** 2021-05-06

**Authors:** Meshari Attar, Yaser M Alsinnari, Mohammed S Alqarni, Ziad M Bukhari, Abdulmalek Alzahrani, Abdulkarim W Abukhodair, Ammar Qadi, Maryam Alotibi, Nisreen A Jastaniah

**Affiliations:** 1 Medicine, King Saud Bin Abdulaziz University for Health Sciences, Jeddah, SAU; 2 Internal Medicine, College of Medicine, King Saud Bin Abdulaziz University for Health Sciences, Jeddah, SAU; 3 Research, College of Medicine, King Saud Bin Abdulaziz University for Health Sciences, Jeddah, SAU; 4 Geriatrics, King Abdulaziz Medical City, Ministry of National Guard - Health Affairs, Jeddah, SAU

**Keywords:** elderly, fall, fractures, occupational therapy, physical therapy

## Abstract

Introduction

Falls in elderlies are one of the leading causes of emergency visits worldwide. It is also a major cause of morbidity and mortality and imposes a significant burden on health care costs. This study investigates risk factors in elderlies aged 65 and above that contribute to falls.

Methodology

This study is a cross-sectional study using a non-probability consecutive sampling technique. The records of 300 clinical data of elderly who underwent falls were collected from all confirmed cases of falls from January 2015 to January 2020, at National Guard Hospital in Jeddah, Kingdom of Saudi Arabia.

Results

Patients included in this study were ranged in age from 65 to 85 years with a mean age of 77.6 years (SD = 8.1 years). Among our population, 149 (53.4%) were males, and 130 (46.6%) were females. Some comorbidities were associated with our population such as diabetes mellitus (69.2%, n = 193), hypertension (75.3%, n = 210), smoking (6.1%, n = 6.1), and polypharmacy (18.3%, n = 51).

Conclusion

Understanding and evaluating risk factors can help to decrease or even prevent falls. Smoking and dementia are strongly related to increased mortality rate. Some outcomes of falls such as head injuries and ICU admission had a strong association to increased mortality. Physical therapy or occupational therapy found to be a strong factor to decrease fall recurrence.

## Introduction

Falling is considered a common incidence that every person encounters throughout their life without much thought. Although this is usually the case for most people, it is a very dangerous process for the elderly. Old age brings inevitable physical changes to the human body, which is problematic. Compromised physical power, muscle weakness, visual and auditory problems, compression fractures of the vertebra, and weakness of back muscles are the major risk factors associated with falling in the elderly population [1].

Falls in the elderly population are a major contributor to morbidity as well as mortality in older individuals. According to the Center for Disease Control and Prevention (CDC), falls are the most common cause of traumatic brain injuries among the elderlies [2]. Falls have a significant impact on elderly to the point where it is the primary determinant and factor in causing the hospitalization of patients with dementia [3]. Recurrent falling is also considered an indicator of poor physical health and poor cognitive ability of the person [4]. A study showed that 357 individuals reported some causes of falls such as syncope, stroke, epilepsy, metabolic disorder, drop attack, psychogenic attack, unexplained falls, autonomic disturbances, orthostatic hypotension, and dementia.

Understanding falls in elderly population is critical as it is responsible for 60% of head injuries and an alarming 90% of the overall wrist and hip injuries [5]. A study conducted to correlate between fear of falling and its consequence on activity reported a prevalence of fear of falling in elderly to be nearly 50%, and around 65% of them reported activity restriction [6]. Falls exhibit a direct negative impact on the elderly, but activity restriction due to fear of falling can cause indirect negative consequences such as weakened muscles and posture, which will ultimately lead to falling feeding a vicious cycle. Since every major activity such as sitting, standing, and walking, contribute to the causation of falls; therefore, various clinical instruments must be incorporated to assess and analyze the risks of falls in elderly patients [6].

Clinical instruments are inaccurate in assessing the risk of falling due to patients’ physical and cognitive impairment [5]. Hence, this study aims to determine and evaluate the common types of falls in the elderly population and their associated risk factors at King Abdulaziz Medical City in Jeddah, Saudi Arabia.

## Materials and methods

This study is a cross-sectional study using a non-probability consecutive sampling technique. The records of 300 clinical data of elderly who underwent falls were collected from all confirmed cases of falls from January 2015 to January 2020, at National Guard Hospital in Jeddah, Kingdom of Saudi Arabia. The inclusion and exclusion criteria were retrospectively reviewed on soft files (BestCare). The inclusion criteria were all elderly patients over 65 years old who have been diagnosed with any type of falls, and any patients aged less than 65 years were excluded. The research started by gathering 300 patients’ soft files, and patients were excluded by fulfilling the exclusion criteria. A total of 283 patients were included in this study. A table was constructed which included the following demographics: age, gender, BMI, past medical history, living condition, date of diagnosis, vital signs on admission, falling history, the outcome of fall, and date of last follow-up. The research table also contained patient outcomes like a referral to occupational or physical therapy, modification of the medication, recurrence of falls, and mortality. All patients’ information was gathered and then placed into the table accordingly.

All data collected were entered and analyzed using IBM Statistical Package for the Social Sciences (SPSS) version 23 (IBM Corp., Armonk, NY). Numerical values and demographic data gathered from the data sheet were computed and presented by frequency and percentage. In addition, a data mean was calculated, and all numerical findings were systematically arranged to provide a quantitative data summary and descriptive data analysis. Independent variables with P value <0.05 will be considered significant.

## Results

Our sample size included 279 patients. Patients included in this study were ranged in age from 65 to 85 years with a mean age of 77.6 years (SD = 8.1 years). Among our population, 149 (53.4%) were males, and 130 (46.6%) were females. Some comorbidities were associated with our population such as diabetes mellitus (69.2%, n = 193), hypertension (75.3%, n = 210), smoking (6.1%, n = 6.1), and polypharmacy (18.3%, n = 51). Demographics and other comorbidities are mentioned in Table 1.

**Table 1:** Demographic characteristics - Medical history & clinical assessment of patients (N = 279) SD: Standard deviation; BMI: Body mass index

Variables	N (%) or mean + SD
Demographic Characteristics	
Gender	
Male	149 (53.4%)
Female	130 (46.6%)
Age (years)	77.6 + 8.1
Weight (kg)	67.5 + 16.7
Height (m^2^)	1.57 + 0.1
BMI (kg/m^2^)	
Underweight	20 (7.2%)
Normal	91 (32.6%)
Overweight	83 (29.7%)
Obese	85 (30.5%)
Medical History	
Smoking	17 (6.1%)
Diabetes Mellitus	193 (69.2%)
Hypertension	210 (75.3%)
Dyslipidemia	87 (31.2%)
Osteoporosis	46 (16.5%)
Polypharmacy	51 (18.3%)
Ischemic heart disease	50 (17.9%)
Heart failure	19 (6.8%)
Atrial fibrillation	11 (3.9%)
Brain tumor	4 (1.4%)
Alzheimer's disease	6 (2.2%)
Dementia	11 (3.9%)
Cerebrovascular accident	39 (14%)
Parkinson's disease	9 (3.2%)
Hypothyroidism	30 (10.8%)
Psychiatric disorders	16 (5.7%)
History of fall	52 (18.6%)
Use of walking aid	58 (20.8%)
History of previous surgeries	93 (33.3%)
Living conditions	
With family	272 (97.5%)
Alone	7 (2.5%)

Vital signs upon admission included heart rate (HR), oxygen saturation (O2 sat), blood pressure (BP), respiration rate (RR), and temperature. For HR most patients presented with normal HR (85.7%, n = 239) followed by tachycardia and bradycardia (11.8%, n = 33; 2.5%, n = 7), respectively. Patients had O2 sat mean of 96.8 with SD of 3.2. For BP, the most common presentation was hypertension (79.2%, n = 221), followed by normal BP and hypotension (20.1%, n = 56; 0.7%, n = 2), respectively. RR had a mean of 20.1 BPM with SD of 2.6, and lastly, the temperature mean was found to be 36.8 with SD of 0.4. Fractures were seen in 82.4% of patients (n = 230), and the most common type of fractures in elderlies after falling was found to be hip fracture (47%, n = 131) followed by femur (6.1%, n = 17), humerus (5.7%, n = 16), and ankle (4.7%, n = 13). All types of fractures are listed in Table 2.

**Table 2:** Types of fractures

Fractures	(n = 230)
Cervical	4 (1.4%)
Humerus	16 (5.7%)
Ribs	8 (2.9%)
Lumber	12 (4.3%)
Radius	7 (2.5%)
Ulnar	3 (1.1%)
Femur	17 (6.1%)
Tibial	6 (2.2%)
Metacarpal Bones	3 (1.1%)
Ankle	13 (4.7%)
Skull	5 (1.8%)
Hip	131 (47%)
Thoracic	5 (1.8%)
Unknown	49 (17.6%)

The most common mechanism of fall in elderly was found to be other (28.3%, n = 79) followed by forward fall (19.4%, n = 54), right side fall (19%, n = 53), left side fall (15.8%, n = 44), backward fall (13.6%, n = 38), and unknown mechanism (3.9%, n = 11). Most common place of fall was found to be home (82.2%, n = 231), followed by outdoor (8.6%, n = 24), hospital (6.1%, n = 6.1), and unknown place (2.5%, n = 7). There was no significant association between risk factors and the mechanism of fall or place of fall.

Our population is presented with a mortality rate of 12.9% (n = 36), and there was no association between risk factors and mortality, except, significant association with smoking and dementia. The association between mortality and the outcomes following the falls is seen in Table 3.

**Table 3:** Comparison between mortality and medical history of patients DM: Diabetes mellitus; IHD: Ischemic heart disease. *chi-square or Fishers’ exact test was used.

Items	Mortality		p-value
	Yes	No	
Smoking	5 (29.4%)	12 (70.6%)	0.036*
No smoking	31 (11.8%)	231 (88.2%)	
DM	25 (13%)	168 (87%)	0.970
No DM	11 (12.8%)	75 (87.2)	
Osteoporosis	5 (10.9%)	41 (89.1%)	0.653
No osteoporosis	31 (13.3%)	202 (86.7%)	
Polypharmacy	5 (9.8%)	46 (90.2%)	0.465
No polypharmacy	31 (13.6%)	197 (86.4%)	
IHD	6 (12%)	44 (88%)	0.833
No IHD	30 (13.1%)	199 (86.9%)	
Dementia	7 (36.6%)	4 (36.4%)	0.018*
No dementia	32 (11.9%)	236 (88.1%)	
History of Fall in the past			0.214
Yes	4 (7.7%)	48 (92.3%)	
No	32 (14.1%)	195 (85.9%)	

Head injury was observed in 10.4% (n = 29) of elderlies following falls. There were 9% (n = 25) with subdural hematoma, 0.7% (n = 7) with intracranial hemorrhage, 0.4% (n = 1) with epidural hematoma and 0.4% (n = 1) with subarachnoid hemorrhage. Also, ICU admission was seen in 4.7% (n = 13), ward admission was seen in 84.9% (n = 237), surgeries was seen in 74.2% (n = 207) and discharge was seen in 16.1% (n = 45).

Elderlies who underwent ICU admission upon falling had the highest mortality rate compared to patients who were not admitted to ICU (38.5%, n = 5) with a significant p-value of 0.016. Second most common fall outcome associated with mortality was found to be head injury (31%, n = 9), with significant p-value of 0.006. Outcome of fall and its association to mortality is illustrated in Table 4.

**Table 4:** Comparing the mortality vs outcome of falls ICU: Intensive care unit *chi-square test was used.

Items	Mortality		p-value
	Yes	No	
Head Injuries	9 (31%)	20 (69%)	0.006*
No Head Injuries	27 (10.8%)	223 (89.2%)	
ICU admission	5 (38.5%)	8 (61.5%)	0.016*
No ICU admission	31 (11.7%)	235 (88.3%)	
Ward admission	30 (12.7%)	207 (87.3%)	0.772
Ward discharge	6 (14.3%)	36 (85.7%)	
Surgeries	27 (13%)	180 (87%)	0.906
No Surgeries	9 (12.5%)	63 (87.5%)	
Fractures	32 (13.9%)	198 (86.1%)	0.276
No fractures	4 (8.2%)	45 (91.8%)	

In interventions implemented for falling, referral to physical therapy was used in 61.6% (n = 172) of patients, and occupational therapy was also used in 57.3% (n = 160) of patients. Recurrence of falling in the future for the overall patients was found in 13.6% of the patients (n = 38). Specifically, elderlies who underwent physical therapy had a low recurrent fall rate of 9.3% (n = 16) compared to patients who were not referred to physical therapy (20.6%, n = 22) with a significant p-value of 0.008. Elderlies who were referred to occupational therapy also had a low recurrence rate of falling (10%, n = 16) vs non-referral to occupational therapy patients with a significant p-value of 0.041. Medication modification was used in four patients and none of them had recurrent falls, and p-value was found to be not significant (p = 0.424). Elderlies who had previous history of falling in the past had a higher rate of fall recurrence (23%) compared to those who fell once (11.5%) with p-value of 0.041, thus the risk of falling is almost double if recurrent fall history was positive. Other medical histories were not significantly associated with recurrence.

## Discussion

Falling in elderly is considered a critical indicator of poor general health and requires prompt urgent evaluation because of its impact on survival rate and mortality. Mechanism of fall and its association with mortality were assessed in our study and it was found to be insignificant with a p-value of >0.05. However, another study has found that falls that involved a long lie afterward had a significant impact on mortality. Out of 20 elderlies who had long-lie fall, 11 of them died compared to those who did not experience a long-lie fall where out of 105 only 21 died [7]. In this study, long-lie and non-long-lie falls were not assessed among mechanisms of falls; as such, its association to mortality was not determined. Among all risk factors assessed, only smoking and dementia were strongly associated with mortality with a p-value < 0.05. Similarly, a study was conducted using population data examining dementia and its association with mortality. The study found a significant correlation to mortality where the 5-year probability of survival of dementia was comparable to those with ischemic heart disease or cancer [8].

In regards to the outcome of falls in our study, we found a strong association between head injuries after falling and mortality. Similarly to our findings, another study found that traumatic brain injury increased hospitalization rates significantly over five years, with advanced age, injury severity, and comorbidity as independent predictors of both mortality and falls [9]. Another outcome of fall which we found in our study to be associated with mortality is ICU admission. Although ICU admission was positively associated with mortality in our study, we could not determine whether it was an admission due to falls or to co-morbidities and general health status.

The only significant association between recurrence of falls and risk factors was a history of previous falls. According to our data, we found that having a history of previous fall increased the chances of falling by double compared to those who fell only once. In contrast to our findings, another study has concluded that the elderly revisit the emergency department frequently due to falling and that within 20 days will revisit due to another fall as well as it being one of the major causes of mortality [10]. They also suggested that emergency physicians should take steps to determine the etiology of the fall rather than only managing fall-related outcomes. It is imperative to determine the etiology of the fall to take measures in management to prevent recurrence. Our study found that elderlies who were referred to either physical therapy or occupational therapy had a very low risk of fall recurrence (13.6%). In contrast to our findings, a study concluded that physical exercise intervention can significantly reduce fall rate and risk [11]. Although we had similar findings, this study examined healthy elderly, which is different from our study population with multiple comorbidities.

Although fall-related injuries are not a common cause of death in the elderly, accidental falls due to unknown causes are among the top causes of unintentional mortality in those aged 65 and above. Death related to falls increases with advancing age and a greater number of comorbidities. Certain fall-related injuries, such as hip fractures, are associated with high mortality, especially in men more than women. Our study has many limitations, it is retrospective cross-sectional study and it is a single-center study, which may affect generalizability of its conclusion. Another limitation is the small number of patients and a significant number of our patients were excluded due mainly to incomplete clinical data and/or follow-up, making interpretation of the risk factor of mortality limited. Another limitation encountered was the lack of data to correlate between time of fall and hospital admission to mortality and morbidity.

## Conclusions

Elderly individuals among the population of 65 years or over have a high tendency to fall due to either intrinsic or extrinsic factors. Falling in elderlies is a very common cause of injuries that increase the rate of mortality and significantly decrease quality of life. There are many predisposing factors that may lead to falls. Understanding and evaluating risk factors can help to decrease or even prevent falls. Smoking and dementia are strongly related to increased mortality rate. Some outcomes of falls such as head injuries and ICU admission had a strong association with increased mortality. Physical therapy or occupational therapy found to be a strong factor to decrease fall recurrence. Knowing the main common mechanism of fall can aid health care providers in their approach in order to treat and prevent dangerous outcomes of falls such as fractures. Future researches are needed to view whether specific fall mechanisms lead to deadly fractures and how such falls can be prevented.
